# TCR-CD3 signal strength regulates plastic coexpression of IL-4 and IFN-γ in Tfh-like cells

**DOI:** 10.3389/fimmu.2024.1481243

**Published:** 2024-11-08

**Authors:** Niels J. M. Verstegen, Tineke Jorritsma, Anja ten Brinke, Matteo Barberis, S. Marieke van Ham

**Affiliations:** ^1^ Department of Immunopathology, Sanquin Research and Landsteiner Laboratory, Amsterdam UMC, University of Amsterdam, Amsterdam, Netherlands; ^2^ Synthetic Systems Biology and Nuclear Organization, Swammerdam Institute for Life Sciences, University of Amsterdam, Amsterdam, Netherlands; ^3^ Molecular Systems Biology, School of Biosciences, Faculty of Health and Medical Sciences, University of Surrey, Guildford, United Kingdom; ^4^ Centre for Mathematical and Computational Biology (CMCB), University of Surrey, Guildford, United Kingdom; ^5^ Swammerdam Institute for Life Sciences, University of Amsterdam, Amsterdam, Netherlands

**Keywords:** TCR signaling, Tfh cell, cytokine plasticity, IL-21, T cell differentiation

## Abstract

The development of T follicular helper (Tfh) cells is an ongoing process resulting in the formation of various Tfh subsets. Despite advancements, the precise impact of T cell receptor (TCR) stimulation on this process remains incompletely understood. This study explores how TCR-CD3 signaling strength influences naive CD4^+^ T cell differentiation into Tfh-like cells and the concurrent expression of interleukin-21 (IL-21), interleukin-4 (IL-4), and interferon-gamma (IFN-γ). Strong TCR-CD3 stimulation induces proliferation and increased IL-21 expression in Tfh-like cells, which exhibit a characteristic phenotype expressing CXCR5 and PD1. The coexpression of IL-4 and IFN-γ in IL-21-producing Tfh-like cells is controlled by the strength TCR-CD3 stimulation; low stimulation favors IL-4, while strong stimulation enhances IFN-γ secretion. Exogenous addition of the effector cytokines IL-21 and IL-4 further modulate cytokine coexpression. These findings highlight the intricate regulatory mechanisms governing cytokine production and plasticity in Tfh-like cells, providing insights into B cell response modulation. *In vivo*, antigen availability may regulate Tfh cell plasticity, impacting subsequent B cell differentiation, emphasizing the need for further exploration through animal models or antigen-specific Tfh cell analyses in human lymph node biopsies

## Introduction

CD4^+^T cells are vital in the adaptive immune response against pathogens and foreign substances. Dendritic cells (DCs) capture antigens and present them as peptide-MHCII complexes on their cell membrane. In secondary lymphoid organs like lymph nodes, naive CD4+ T cells engage with these complexes through their T cell receptors (TCRs) (1). Upon activation, naive CD4^+^T cells undergo division and differentiation into various T helper (Th) cell subsets, influenced by signals from DCs (2, 3). Th cell subsets are acknowledged to possess plasticity, allowing them to adapt and respond to diverse immune challenges (4–9).

DC activation can induce the differentiation of a subset of CD4^+^ T cells known as pre-T follicular helper (Tfh) cells (10). These transient cells differentiate into Tfh cells, playing a pivotal role in assisting B cells in the immune response (11). Following activation, pre-Tfh cells engage with activated B cells in the T-B cell interface, a crucial step for their differentiation into Tfh cells (12, 13). Tfh cells express specific markers like CXCR5 and PD1, enabling them to enter B cell follicles and engage in the germinal center (GC) reaction (14). Within the GC, Tfh cells facilitate affinity-based selection, resulting in the generation of memory B cells and antibody-secreting cells. The regulatory mechanisms governing this process include Tfh-derived cytokine plasticity (15). Interestingly, deficiency in SNARE proteins that facilitate vescicle fusion (16) and in metabolic regulations (17) that modulate T‐ and B‐cell interaction leads to an impaired GC formation and T cell–dependent B cell responses underlying hyperinflammatory syndrome and pro‐inflammatory states occurring in autoimmune disorders, respectively.

Despite extensive research on B cell selection in the GC, the dynamics of Tfh cell behavior remain incompletely understood. Antigen availability emerges as a potential regulator, given that interactions with GC B cells increase intracellular calcium and stimulate cytokine production in Tfh cells (18). Additionally, increased antigen presentation by GC B cells leads to enhanced TCR-CD3-related gene expression and proliferation in Tfh cells (19). In the classical Th1/Th2 polarization, strong TCR-CD3 signaling favors Th1 (IFN-γ) polarization, while weak signaling promotes Th2 (IL-4) polarization (20–23). Tfh cells were initially thought to require strong TCR-CD3 signaling, but recent research suggests they can develop within a range of signaling strengths (24–31). This manuscript explores the regulation of IL-4 and IFN-γ coexpression in IL-21-producing Tfh-like cells by TCR-CD3 signaling strength.

## Materials and methods

### Purification of CD4^+^ T cells

Human peripheral blood mononuclear cells (PBMC) were isolated through standard gradient centrifugation using Ficoll-lymphoprep (Axis-Shield) from buffy coats obtained from healthy blood donors (Sanquin Blood Supply). Donors gave informed consent approved by the local institutional review board and the Medical Ethics Committee of Sanquin Blood Supply. CD4^+^ T cells were purified from PBMCs using anti-CD4 Dynabeads and DETACHaBEAD (Invitrogen). Untouched naive CD4^+^ T cells (CD4^+^CD45RO^-^) were isolated with high purity (>98%) using CD45RO-PE antibodies and anti-PE beads (MACS; Miltenyi Biotec). Cells were cryopreserved in liquid nitrogen.

### Naive CD4^+^ T cell stimulation

Naive CD4^+^ T cells labeled with Cell Trace CFSE according to manufacturer’s instructions (Invitrogen) were cultured in 96-round bottom well plates at a density of 2.5 x 10^3^/well in a total volume of 200 µl complete RPMI 1640 medium (Invitrogen), supplemented with 5% FCS (Bodinco), 100 U/ml penicillin (Invitrogen), 100 µg/ml streptomycin (Invitrogen), 2 mM L-glutamine (Invitrogen), 50 µM 2-ME (Sigma) and 20 µg/ml human apotransferrin (Sigma; depleted for human IgG with protein G Sepharose (Amersham Biosciences)). Cells were activated with varying doses of anti-CD3 (clone 1XE; Sanquin) and 1 μg/mL anti-CD28 (clone 15E8; Sanquin) with or without 50 ng/mL IL-21 (Invitrogen), 50 ng/mL IL-4 (Cellgro), and 50 ng/mL IFN-γ (Peprotech).

### Flow cytometry analysis

Surface markers and intracellular cytokines were detected by the following FACS anti-human antibodies: CD4 (SK3; BD Biosciences); IL-21 (3A3-N2; eBiosciences); IFN-γ (B27; BD Biosciences); and IL-4 (3010.211; BD Biosciences). Before staining, cells were stimulated in a complete medium with 0.1 µg/ml PMA (Sigma), 1 µg/ml ionomycin (Sigma), and 10 µg/ml brefeldin A (Sigma) for 5 hours. Samples were washed twice with PBS and stained with a Fixable Near-IR Dead Cell Stain Kit (Invitrogen). Cells were fixed with 4% PFA for 15 minutes, permeabilized with 0.5% saponin in PBS containing 1% BSA, and incubated with fluorescent antibodies for 30 minutes at room temperature. All cells from the samples were collected on an LSRII flow cytometer (BD Bioscience) and analyzed with FACSDiva software (BD) and FlowJo version 10 (Treestar).

### RNA isolation and qRT-PCR

RT-PCR was performed as previously described (32). RNA was reverse transcribed to cDNA using random hexamers, Superscript II, and an RNase H-reverse transcriptase kit. Primers for 18S rRNA, IL-21, CXCR5 and BCL6 were designed to prevent genomic DNA amplification (**Supplementary Table 1**). Gene expression levels were measured in triplicate using SYBR green method in StepOnePlus (Applied Biosystems), normalized to 18S rRNA as the internal control.

### Statistical analysis

Statistical analyses were performed using Prism 9 (Graphpad). The statistical tests used are indicated in the figure descriptions. Data show the mean of multiple donors, and error bars represent mean ± SEM.

## Results

### High TCR-CD3 stimulation facilitates the induction of IL-21-expressing Tfh-like cells

**Figure 1:**
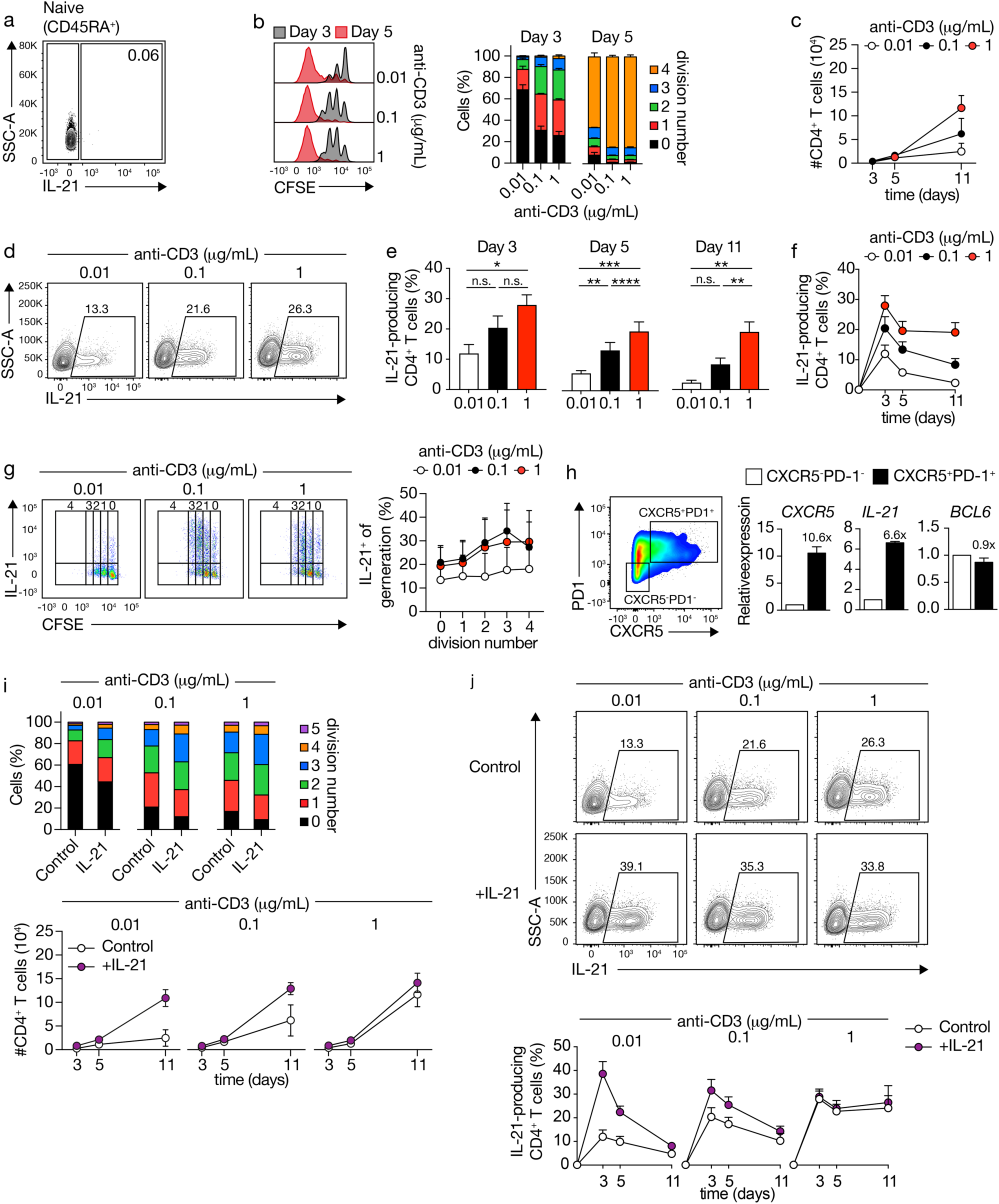
CD4^+^ T cells produce IL-21 and acquire a Tfh-like phenotype upon increasing TCR-CD3 stimulations. **(A)** IL-21 expression in human naive CD4^+^ T cells analyzed by flow cytometry. **(B)** CFSE-labeled CD4^+^ T cells stimulated with varying concentrations of anti-CD3 antibodies for 3 or 5 days (left), graph of the cell division fraction (right). **(C)** Live CD4^+^ cell counts after TCR-CD3 stimulation. **(D)** IL-21 expression in CD4^+^ T cells after 5 days of TCR-CD3 stimulation. Cell percentages are indicated in the gate. **(E, F)** Quantification of IL-21 production after TCR-CD3 stimulation for 3 (n=6), 5 (n=17), and 11 (n=6) days. **(G)** CFSE-labeled CD4^+^ T cells stimulated with anti-CD3 for 3 days, with fraction of IL-21-positive cells at each division. **(H)** CD4^+^ T cells at day 3 after stimulation with 1 μg/mL TCR-CD3, with sorted CXCR5^-^PD1^-^ and CXCR5^+^PD1^+^ cells analyzed for *CXCR5*, *IL-21* and *BCL6* mRNA expression (n=2). **(I)** Fraction of CD4^+^ T cells within each division after TCR-CD3 and rIL-21 stimulation for 3 days, and live CD4^+^ cell counts (n=3). **(J)** IL-21 expression after 5 days of TCR-CD3 and rIL-21 stimulation, with quantification for 3, 5, and 11 days. Data analyzed by repeated-measures one-way ANOVA with Sidak post-test; *p < 0.05, **p < 0.01, ***p < 0.001, ****P < 0.0001, n.s., not significantly different. Stacked bars represent mean (n=3) of individual donors.

We examined the impact of T cell receptor (TCR) stimulation strength on naive CD4^+^ T cell differentiation into IL-21-secreting Tfh-like cells. It is noteworthy that under basal conditions, IL-21 expression remains undetectable in human naïve CD4+ T cells (**Figure 1A**). These cells, subjected to varying doses of CD3 and optimal CD28 stimulation (1 μg/mL), exhibited increased proliferation and live cell numbers, particularly evident at later time points (**Figures 1B, C**). Analysis of IL-21 production at distinct time intervals revealed an anti-CD3 dose-dependent induction of IL-21 expression, peaking shortly (3 days) after TCR-CD3 stimulation (**Figures 1D–F**). Accordingly, IL-21 production occurred early during cell division, indicating a prompt response to TCR-CD3 stimulation (**Figure 1G**).

To ascertain whether IL-21-expressing cells exhibited a Tfh-like phenotype, we evaluated CXCR5 and PD1 expression (**Figure 1H**). Stimulation with PMA/Ionomycin, supplemented with Brefeldin A, notably reduced CXCR5 expression, consistent with previous findings (33) (data not shown). To evaluate IL-21 expression and other Tfh characteristics, we isolated CXCR5^-^PD1^-^and CXCR5^+^PD1^+^populations during the peak of IL-21 production (3 days) induced by robust TCR-CD3 stimulation. Subsequent analysis of *CXCR5* and *IL-21* mRNA expression in both populations revealed a higher expression in CXCR5^+^PD1^+^ compared to CXCR5^-^PD1^-^ CD4^+^ T cells, whereas the expression of *BCL6* was similar, confirming the Tfh-like phenotype of IL-21-secreting cells (**Figure 1H**).

The formation of Tfh cells depends on a CD4^+^ T cell-intrinsic requirement for IL-21 (34). To determine the autocrine or paracrine effects of IL-21 during CD4^+^ T cell differentiation, exogenous IL-21 was introduced, leading to enhanced proliferation and division rates, particularly under low TCR-CD3 stimulation (**Figure 1I**). Moreover, the strength of TCR-CD3 signaling dictated the capacity of exogenous IL-21 to augment the differentiation of IL-21-producing CD4^+^ T cells. In fact, notably, the percentage of induced IL-21-producing cells through TCR-CD3 stimulation reached a plateau, beyond which supplementary IL-21 failed to further enhance differentiation (**Figure 1J**). In summary, heightened TCR-CD3 stimulation induces IL-21 in Tfh-like cells. Conversely, lower stimulation yields reduced intracellular IL-21, but exogenous IL-21 can enhance production, suggesting an intricate regulatory mechanism involving autocrine or paracrine signals.

### The plastic coexpression of IL-4 and IFN-γ by IL-21-producing Tfh cells requires a delicate balance in TCR-CD3 stimulation

The plasticity of Tfh cells in expressing cytokines with diverse effects on GC responses and memory B cell or antibody-secreting cell formation remains unclear. We investigated the impact of TCR-CD3 signal strength on IFN-γ and IL-4 expression as these are the main cytokines expressed by Tfh cells known to help B cell differentiation. Consistent with the Th1 and Th2 dogma (20–23), low-intensity TCR-CD3 stimulation facilitated IL-4 production at day 5, gradually decreasing thereafter (**Supplementary Figures 1A, B**). Intermediate and high stimulations initially induced fewer IL-4-producing cells, with proportions increasing later (**Supplementary Figure 1B**). Strong TCR-CD3 stimulation resulted in substantial IFN-γ production (**Supplementary Figures 1C, D**). Unlike IL-21, IL-4 and IFN-γ secretion correlated strongly with cell division, increasing progressively over divisions (**Supplementary Figures 2E, F**). Exogenous IL-4 and IFN-γ provided positive feedback, particularly for IL-4 under low TCR-CD3 stimulation (**Supplementary Figures 1E, F**).

**Figure 2:**
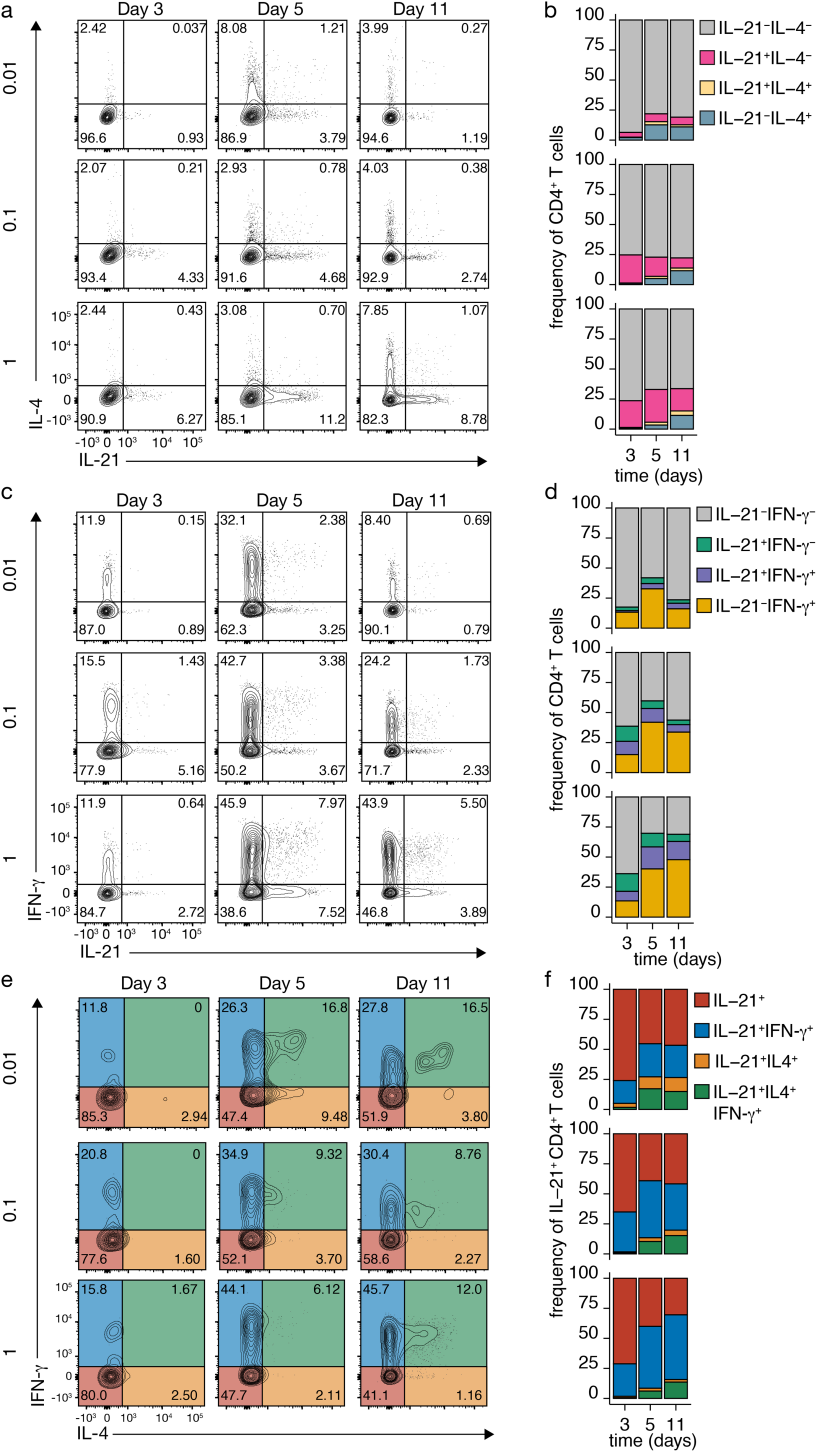
Dynamic Cytokine Expression in CD4^+^ T Cells upon TCR-CD3 Stimulation. **(A-D)** IL-4 and IL-21 **(A, B)** or IFN-γ and IL-21 **(C, D)** co-expression in CD4^+^ T cells after variable TCR-CD3 stimulation. **(E, F)** IL-4 and IFN-γ co-expression in IL-21-producing Tfh-like cells after different anti-CD3 concentrations. Stacked bars represent mean (n=3) individual donors.

We investigated whether IL-21-producing Tfh-like cells could coexpress IL-4 and IFN-γ through varying TCR-CD3 signaling strength (**Figures 2A–D**). Naive CD4^+^ T cell priming mainly yielded cells expressing either IL-4 or IL-21, with only a small fraction coexpressing both across TCR-CD3 stimulations (**Figure 2B**). However, variation in TCR-CD3 signaling induced more coexpression of IL-21 and IFN-γ (**Figures 2C, D**), especially under strong stimulation (**Figures 2C, D**).

To assess cytokine coexpression in more detail, IL-4 and IFN-γ production were analyzed after TCR-CD3 stimulation in IL-21-producing Tfh-like cells (**Figures 2E, F**). On day 3, most Tfh-like cells produced only IL-21, but coexpression of IFN-γ increased, especially with higher stimulation (**Figures 2E, F**). From day 5 on, the number of IL-21-producing Tfh-like cells coexpressing IL-4 increased, particularly with low TCR-CD3 stimulation (**Figures 2E, F**). In these conditions, most cells coexpressing IL-4 also expressed IFN-γ (**Figures 2E, F**). Coexpression of IL-4 alone was mainly observed with low TCR-CD3 stimulation, potentially due to positive feedforward signaling by IL-4 and limited secretion of IFN-γ and IL-21. These findings highlight the complex interplay of cytokines within the CD4^+^ T cell network, with possible positive and negative feedback loops regulating their expression. While single IL-21 expression is observed early after naive CD4^+^ T cell priming, plastic coexpression of IL-4 and IFN-γ occurs at later time points, especially with restricted IL-21 and IL-4 coexpression under low TCR-CD3 stimulation.

### Exogenous cytokines IL-21 and IL-4 regulate plastic coexpression of IL-4 and IFN-γ in IL-21-producing Tfh-like cells

**Figure 3:**
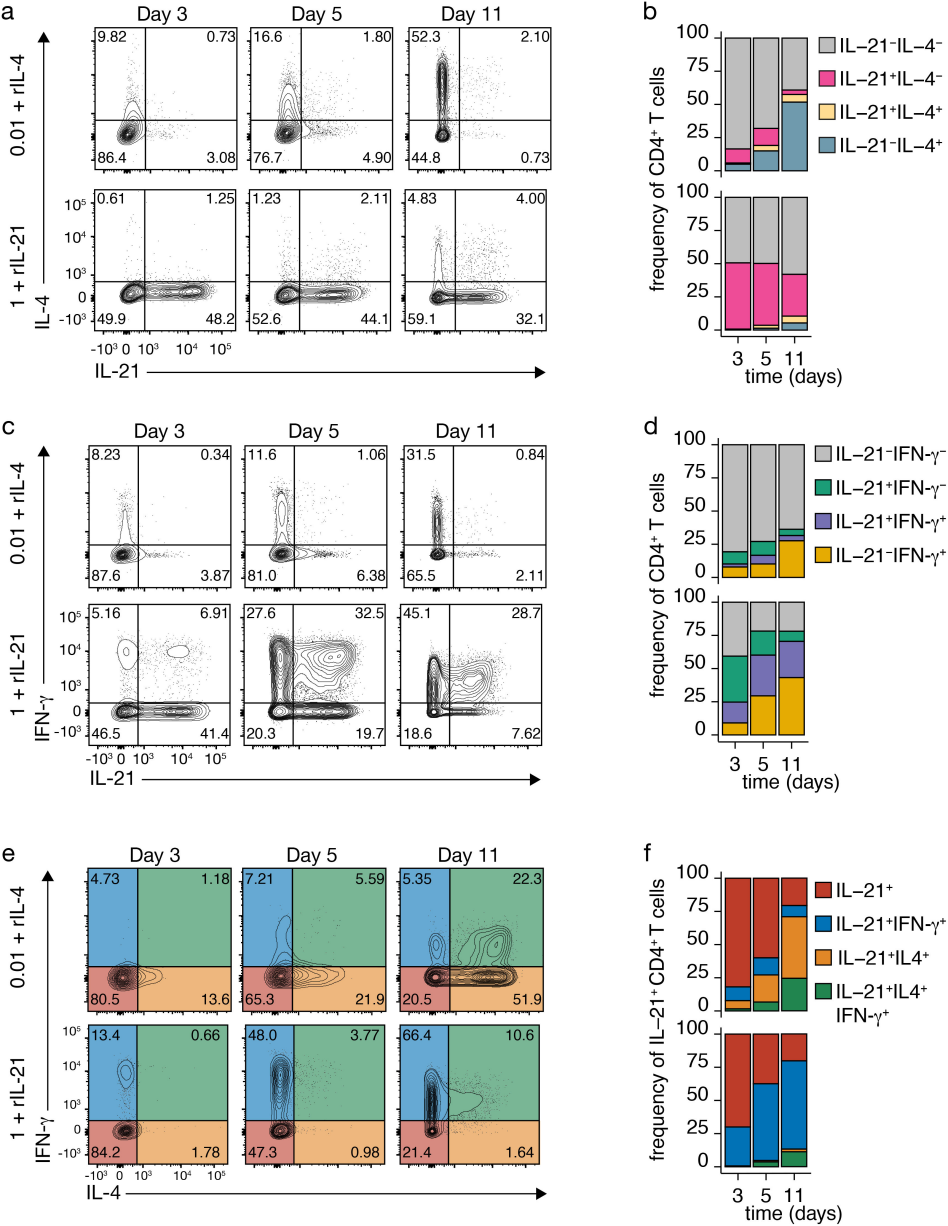
Impact of Recombinant Cytokines on TCR-CD3-Induced Cytokine Expression. **(A-D)** IL-4 and IL-21 **(A, B)** or IFN-γ and IL-21 **(C, D)** co-expression in CD4^+^ T cells after TCR-CD3 stimulation with recombinant IL-4 (rIL-4; top) or rIL-21 (bottom). **(E, F)** IL-4 and IFN-γ co-expression in IL-21-producing Tfh-like cells after TCR-CD3 stimulation with rIL-4 (top) or rIL-21 (bottom). **(F)** Stacked bars represent mean (n=3) individual donors.

The impact of IL-21 and IL-4 on the plastic coexpression of IL-4 and IFN-γ in IL-21-producing Tfh cells was explored, focusing on the effective range of these exogenous cytokines: low TCR-CD3 stimulation for IL-4 and high for IL-21 (**Figure 3**, **Supplementary Figures 2**, **3**). Both IL-4 and IL-21 significantly influenced the coexpression of IL-21 and IL-4, with exogenous IL-4 inducing IL-4 production (**Figures 3A, B** versus **Figures 2A, B**, top rows) and IL-21 inducing IL-21 without concurrent IL-4 expression (**Figures 3A, B** versus **Figures 2A, B**, bottom rows). Interestingly, exogenous IL-21 induced moderate coexpression of IL-21 and IL-4 double-positive cells (**Figures 3A** versus **Figures 2A, B**, bottom rows).

Effects on IL-21 and IFN-γ coexpression were observed (**Figures 3C, D**), with limited coexpression after TCR-CD3 stimulation alone (**Figures 2C, D**), significantly enhanced with exogenous IL-21 (**Figures 3C, D** and **Supplementary Figures 2C, D**), and mainly repressed by exogenous IL-4 (**Figures 3C, D** and **Supplementary Figures 3C, D**). Further analysis of IL-21-producing Tfh-like cells revealed that exogenous IL-4 markedly increased the population coexpressing IL-4 alone or in combination with IFN-γ, particularly after low stimulation (**Figures 3E, F** and **Supplementary Figures 3E, F** versus **Figures 2E, F**, top rows). In contrast, exogenous IL-21 had a minimal impact on IL-4 and IFN-γ coexpression by IL-21-producing cells (**Figures 3E, F** versus 2E,F, bottom rows). Therefore, substantial coexpression of IL-4 by IL-21-producing Tfh cells requires the presence of excess IL-4, potentially acting beyond the immunological synapse, as it has been shown for IL-21 (35).

## Discussion

Our study highlights the pivotal role of TCR-CD3 signaling strength in shaping the differentiation and cytokine plasticity of naive CD4+ T cells into T follicular helper (Tfh)-like cells. We observed that strong TCR-CD3 stimulation drives robust IL-21 and IFN-γ production, whereas lower signaling favors IL-4 expression. This variation in cytokine output indicates that Tfh-like cells are not restricted to a binary Th1/Th2 model but display a flexible, context-dependent functional spectrum.

The induced Tfh-like cells exhibited high levels of *CXCR5* and *IL-21* mRNA, whereas *BCL6*, the central transcription factor regulating Tfh gene expression, showed a comparable expression profile in CXCR5^+^PD1^+^ cells compared to CXCR5^-^PD1^-^ CD4^+^ T cells (36) The upregulation of BCL6 likely requires additional signals beyond CD3 and CD28 stimulation, with interaction with B cells being a key factor. Specifically, Tfh cell-B cell interactions, including T cell receptor (TCR) engagement with antigen-presenting B cells and co-stimulatory signals such as ICOS and CD40L, are critical for the full differentiation of Tfh cells and BCL6 expression. These signals help sustain BCL6 expression, which is essential for the complete differentiation of Tfh cells and the maintenance of their identity and functions (13).

The strong correlation between signal strength and cytokine expression, particularly the coexpression of IL-4 and IFN-γ within IL-21-producing Tfh-like cells, emphasizes the dynamic nature of Tfh cell responses. These findings suggest that Tfh cells are not rigidly polarized but can occupy intermediate states. Moreover, our results show that exogenous cytokines, such as IL-21 and IL-4, can further modulate Tfh cell function. While IL-21 promotes its own expression and limits IL-4, exogenous IL-4 enhances IL-4 production and supports the coexpression of IL-4 and IFN-γ, particularly under low TCR-CD3 stimulation. This suggests that external cytokine environments can fine-tune Tfh cell plasticity, potentially impacting B cell responses and GC dynamics. For example, the interplay of IL-4 and IFN-γ within Tfh-like cells may influence B cell fate, including memory B cell or plasma cell differentiation, depending on the strength of TCR-CD3 signaling (37).

The implications of these findings extend beyond basic immunology and could be leveraged for therapeutic interventions. By manipulating Tfh plasticity through controlled antigen exposure or cytokine supplementation , it may be possible to enhance vaccine efficacy or manage autoimmune diseases by directing specific B cell outcomes.

From a translational perspective, the *in vivo* relevance of these findings is crucial. In physiological settings, the plasticity of Tfh-like cells may allow the immune system to tailor responses to different pathogens or antigenic environments, fine-tuning both the quality and magnitude of B cell-mediated immunity. For instance, during infections, the ability of Tfh cells to adopt intermediate cytokine profiles could help balance the need for protective immunity with the prevention of excessive immune activation. Conversely, in autoimmune diseases, dysregulated TCR signaling and cytokine environments might skew Tfh responses toward pathological outcomes, such as promoting the production of autoantibodies. Understanding these *in vivo* dynamics will be critical for developing interventions aimed at correcting aberrant Tfh activity in autoimmune contexts.

Future studies using *in vivo* models, such as cytokine reporter mice, alongside detailed *ex vivo* analyses of antigen-specific Tfh cells, will be essential to validate these mechanisms in physiological and disease settings (38–40). This will not only deepen our understanding of Tfh cell function in health and disease but could also inform strategies to manipulate Tfh cells for therapeutic benefit.

## Data Availability

The raw data supporting the conclusions of this article will be made available by the authors, without undue reservation.

## Supplementary material

Supplementary Figure 1 Modulating Effects of Th1, Th2, and Tfh Cell Cytokines. **(A, B)** IL-4 expression after TCR-CD3 stimulation for 5 days, with quantification for 3, 5, and 11 days. **(C, D)** IFN-γ expression after TCR-CD3 stimulation for 5 days, with quantification for 3, 5, and 11 days. **(E, F)** IL-4- and IFN-γ-positive CD4+ T cells after TCR-CD3 for 3 days, with the fraction at each division. **(G, H)** Quantification of IL-4 production after TCR-CD3 and rIL-4 stimulation for 3, 5, and 11 days, IFN-γ production after TCR-CD3 and rIFN-γ stimulation for 5 days. Data analyzed by repeated-measures one-way ANOVA with Sidak post-test; *p < 0.05, **p < 0.01, ***p < 0.001, ****P < 0.0001, n.s., not significantly different.

Supplementary Figure 2 Impact of Recombinant IL-21 (rIL-21) on Cytokine Expression. **(A, B)** IL-4 and IL-21 co-expression in CD4+ T cells after TCR-CD3 stimulation with rIL-21 for indicated days, with quantification. **(C, D)** IFN-γ and IL-21 co-expression in CD4+ T cells after TCR-CD3 stimulation with rIL-21 for indicated days, with quantification. **(E, F)** IL-4 and IFN-γ co-expression in IL-21-producing Tfh-like cells after TCR-CD3 stimulation with rIL-21, with quantification. Stacked bars represent mean (n=3) individual donors.

Supplementary Figure 3 Impact of Recombinant IL-4 (rIL-4) on Cytokine Expression. **(A, B)** IL-4 and IL-21 co-expression in CD4+ T cells after TCR-CD3 stimulation with rIL-4 for indicated days, with quantification. **(C, D)** IFN-γ and IL-21 co-expression in CD4+ T cells after TCR-CD3 stimulation with rIL-4 for indicated days, with quantification. **(E, F)** IL-4 and IFN-γ co-expression in IL-21-producing Tfh-like cells after TCR-CD3 stimulation with rIL-4, with quantification. Stacked bars represent mean (n=3) individual donors.
