# Supplementary figures and images for: TCR-CD3 signal strength regulates plastic coexpression of IL-4 and IFN-γ in Tfh-like cells
Source: Front Immunol. 2024 Nov 8;15:1481243. doi: 10.3389/fimmu.2024.1481243 (PMC11581847; doi:10.3389/fimmu.2024.1481243)

# Supplementary Figure 1

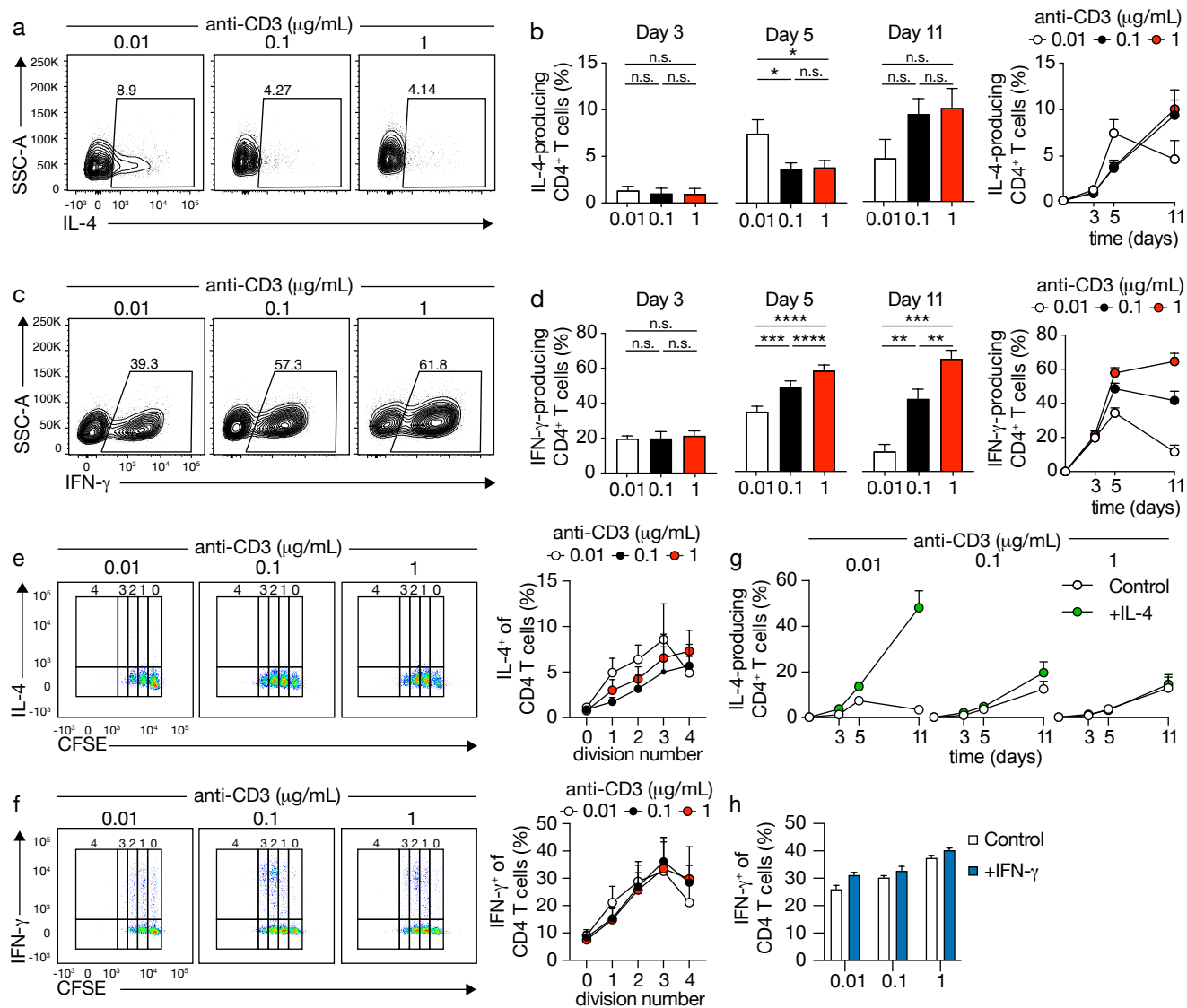

Supplement: Supplementary Figure 1 — Modulating Effects of Th1, Th2, and Tfh Cell Cytokines. (A, B) IL-4 expression after TCR-CD3 stimulation for 5 days, with quantification for 3, 5, and 11 days. (C, D) IFN-γ expression after TCR-CD3 stimulation for 5 days, with quantification for 3, 5, and 11 days. (E, F) IL-4- and IFN-γ-positive CD4+ T cells after TCR-CD3 for 3 days, with the fraction at each division. (G, H) Quantification of IL-4 production after TCR-CD3 and rIL-4 stimulation for 3, 5, and 11 days, IFN-γ production after TCR-CD3 and rIFN-γ stimulation for 5 days. Data analyzed by repeated-measures one-way ANOVA with Sidak post-test; *p < 0.05, **p < 0.01, ***p < 0.001, ****P < 0.0001, n.s., not significantly different. [file Image1.pdf]

# Supplementary Figure 2

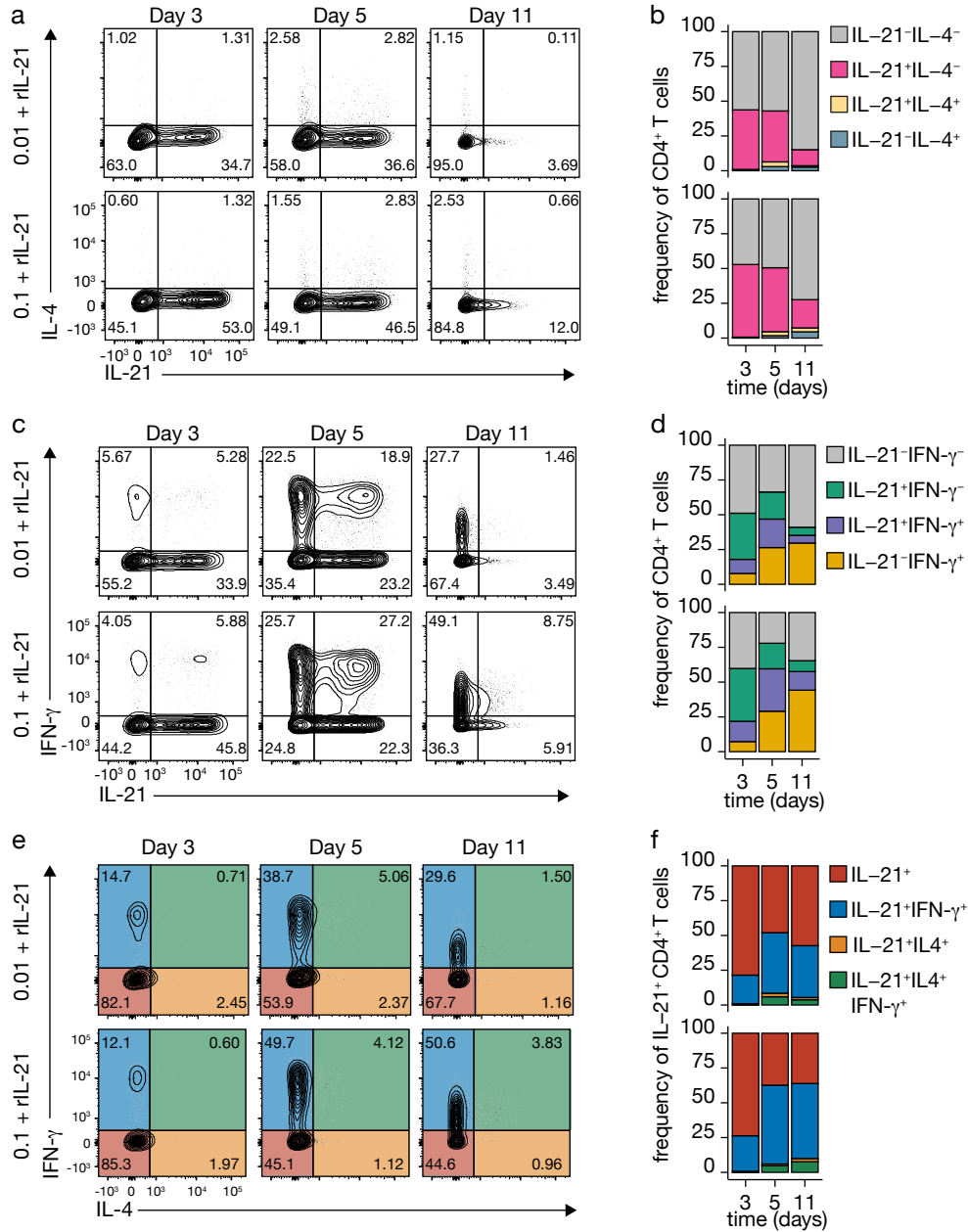

Supplement: Supplementary Figure 2 — Impact of Recombinant IL-21 (rIL-21) on Cytokine Expression. (A, B) IL-4 and IL-21 co-expression in CD4+ T cells after TCR-CD3 stimulation with rIL-21 for indicated days, with quantification. (C, D) IFN-γ and IL-21 co-expression in CD4+ T cells after TCR-CD3 stimulation with rIL-21 for indicated days, with quantification. (E, F) IL-4 and IFN-γ co-expression in IL-21-producing Tfh-like cells after TCR-CD3 stimulation with rIL-21, with quantification. Stacked bars represent mean (n=3) individual donors. [file Image2.pdf]

Supplementary Figure 3

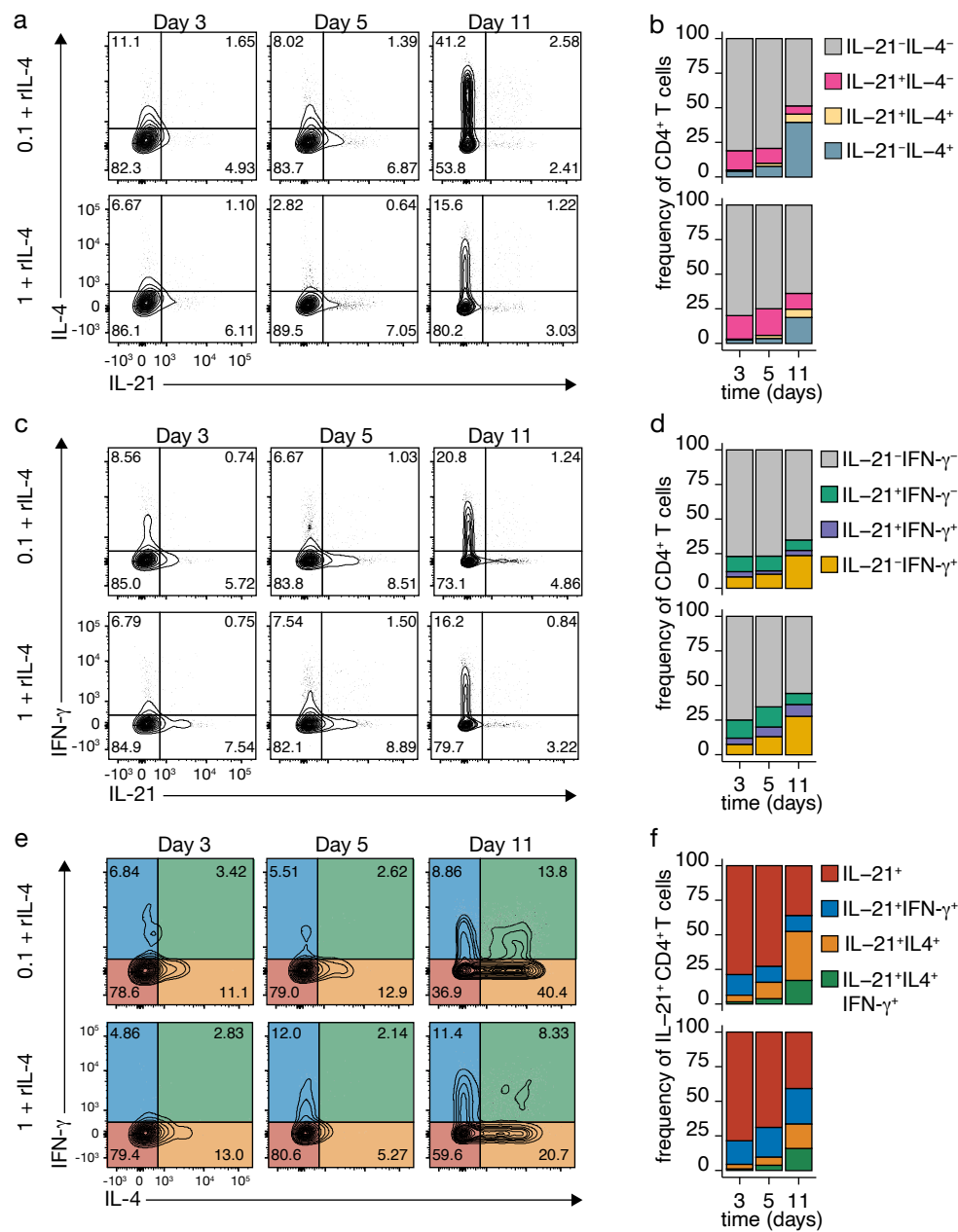

Supplement: Supplementary Figure 3 — Impact of Recombinant IL-4 (rIL-4) on Cytokine Expression. (A, B) IL-4 and IL-21 co-expression in CD4+ T cells after TCR-CD3 stimulation with rIL-4 for indicated days, with quantification. (C, D) IFN-γ and IL-21 co-expression in CD4+ T cells after TCR-CD3 stimulation with rIL-4 for indicated days, with quantification. (E, F) IL-4 and IFN-γ co-expression in IL-21-producing Tfh-like cells after TCR-CD3 stimulation with rIL-4, with quantification. Stacked bars represent mean (n=3) individual donors. [file Image3.pdf]
