# Supplementary material for: TCR-CD3 signal strength regulates plastic coexpression of IL-4 and IFN-γ in Tfh-like cells
Source: Front Immunol. 2024 Nov 8;15:1481243. doi: 10.3389/fimmu.2024.1481243 (PMC11581847; doi:10.3389/fimmu.2024.1481243)
Supplement: Supplementary file 4 [file Table1.docx]

**Supplementary Table 1 | Primer sequences.**

| **Genes** | **Primer** | **Sequence (5’-3’)** |
| --- | --- | --- |
| *18S-rRNA* | Forward  Reverse | CGGCTACCACATCCAAGGAA  GCTGGAATTACCGCGGCT |
| *IL-21* | Forward  Reverse | GTCATCTGTCTGATGGTCATC  CCACTCACAGTTTGTCTCTAC |
| *CXCR5* | Forward  Reverse | CCGCTAACGCTGGAAATGGAC  GCAAAGGGCAAGATGAAGACC |
| *BCL6* | Forward  Reverse | GAGCTCTGTTGATTCTTAGAACTGG  GCCTTGCTTCACAGTCCAA |
